# Adding insult to injury: A review of infections following envenomings

**DOI:** 10.1016/j.toxcx.2025.100230

**Published:** 2025-06-23

**Authors:** Dayle Leonard, Aoife Boyd, Michel M. Dugon

**Affiliations:** a Venom Systems Lab, Ryan Institute, School of Natural Sciences, University of Galway, H91TK33, Galway, Ireland; b Pathogenic Mechanisms Research Group, School of Natural Sciences, University of Galway, H91TK33, Galway, Ireland

**Keywords:** Envenomation, Infection, Snake, Scorpion, Pathogen

## Abstract

Over 100 lineages of animal have evolved venom for a wide variety of purposes. An estimated 2.5 million people are bitten by snakes and another 1.2 million are stung by scorpions with many envenomings resulting in death. However, survival is not the end point of the envenoming syndrome as chronic life altering conditions, such as amputations, disfigurement and neuropathies can occur. In this context, infections at the site of envenomation could play an important role as they can exacerbate mortality and the incidence and severity of life altering conditions.

This review assesses the connection between envenoming and infections. It summarises and highlights the literature describing cases of envenoming-mediated infection by various taxa and the circumstances of these envenomings and the outcomes of infection. It could be deduced that the risk factors for envenoming-led infections are multifactorial. Factors enhancing the risk of infection include; 1. The delivery system, with larger devices leading to more substantial wounds, 2. Venom composition, with venoms containing cytotoxins more commonly implicated in infections, and 3. The environment, with aquatic microbiomes and venom system microbiomes as sources of the pathogen species. Infections are difficult to diagnose due to symptoms synonymous with those of the envenoming and it is recommended that medical practitioners consider the possibility of infection throughout all stages of medical treatment. There is a notable gap in our understanding of envenoming-led infections and further research will help to increase patient survival.

## Graphical abstract

Graphical abstract was produced by availing of Biorender.com.

**Figure:**
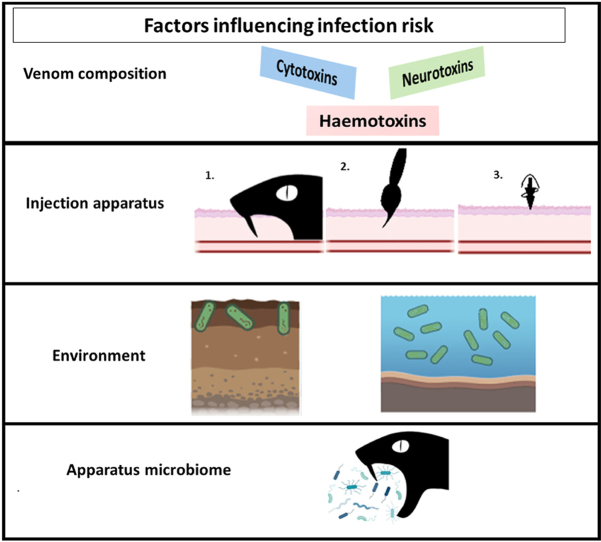

## 1 Introduction

Venom has convergently evolved in over 100 lineages including snakes, spiders, scorpions, bees, sponges, fish, cephalopods and cnidarians (Schendel et al., 2019). Venom evolved to assist with prey capture, defence against enemies and intraspecific competition (Brodie, 2009; White, 2010). Envenoming is typically achieved by a venomous device which overcomes the inability of venom compounds to directly penetrate the skin or cross mucous membranes (Brodie, 2009; Harris and Arbuckle, 2016). These sharp delivery systems typically take the form of stingers, fangs, stinging cells, beaks or spines connected to one or several venom-producing glands.

Venoms act in a combination of three ways. Neurotoxic compounds attack the nervous system causing neuropathies, paralysis and/or death, haemotoxic compounds cause coagulopathy or excess coagulation, inducing stroke or haemorrhaging (Slagboom et al., 2017) and cytotoxic compounds, such as phospholipase A2 (PLA_2_), lyse target cells and are associated with necrotic lesions (Tasoulis and Isbister, 2017). In some cases, envenoming-associated necrotic wounds may result from necroptosis activated in the presence of venom rather than being a direct lytic effect of the venom (Dunbar et al., 2019). The puncture wounds caused by the venom apparatus during envenoming may become the site of bacterial infections (Garg et al., 2009) which in extreme cases may require debridement or amputations (Abubakar et al., 2010; Elserafy, 2023; Kallel et al., 2018).

Whether a venom system may act as a primary reservoir and/or a vector of pathogenic bacteria has been subject to a degree of controversy. While certain authors propose that venom, akin to other bodily fluids, is sterile (Blaylock, 1999; Goldstein et al., 1979; Moreau, 2013), some suggest that the piercing device may act as a vehicle for the inoculation of bacteria (Esmaeilishirazifard et al., 2022), and others propose that venom has antimicrobial properties, thus largely limiting the risk of direct infections (Talan et al., 1991). The latter view has been recently challenged, as evidence of microbes within/on the venom system of animals has surfaced (Dunbar et al., 2020b; Esmaeilishirazifard et al., 2022; Gkitsaki et al., 2023), including the occurrence of antibiotic resistant pathogenic bacterial species (Dunbar et al., 2020b). The association of a unique microbial flora with a venom apparatus increases the risk of infection and may have a direct impact on the symptomatic and final outcomes of the envenoming. In the case of the cobra *Naja nigricollis*, 20 % of the microbiota within the venom system differs to those of the mouth, thus indicating the influence of each microenvironment on microbial community diversity.

The aim of this review is to collate and organise the literature linking envenoming and bacterial infections, in an effort to decipher (1) if venomous bites and stings by specific organisms may increase the likelihood of infection, (2) which pathogens are involved in such events and (3) if any specific envenoming scenarios are more likely to result in subsequent infections.

## 2 Methods

NCBI Pubmed and Google Scholar databases were screened using combinations of the following key search terms: “Bite”, “infection”, “sting”, “envenoming”, “envenomation”, “necrosis”, “amputation” and “case ± study”. These terms were used in conjunction with each other and with taxa names, both vernacular and scientific. Publications were searched regardless of their year of publication. For each publication, the bibliography was screened for further potential publications of interest. Studies were excluded if the animal responsible was not observed by the alleged victim during the envenoming (e.g. in several cases of presumed spider bites). Publications in languages other than English were translated using Google Translate. Papers were accumulated irrespective of bite/sting outcome to avoid bias. 609,998 papers were returned from search combinations, of which 608 publications were read and 205 of those were retained for this manuscript. A breakdown of these papers and associated search terms can be found in Supplementary Table 1.

Publications were collected between September 2023 and July 2024. Publications were grouped based on taxa. We made the choice to consider all marine taxa as a single category. In cases where species were not identifiable, the case was categorised to the highest known taxon level.

## 3 Review

### 3.1 Snakes

The World Health Organisation estimates that 2.5 million people are bitten by venomous snakes every year, resulting in 80,000 to 138,000 deaths and an additional 244,000 to 414,000 victims suffering life altering events, such as amputations (WHO, September 2023). As such, snakes are by far the main contributors to envenoming-associated morbidity worldwide. As of April 2025, there were 4175 species of snakes distributed globally, of which approximately 600 species are venomous (ReptileDatabase, 2025). In the context of snake bites and their medical significance, snakes can be grouped according to their teeth arrangement. Aglyphous snakes (typically pythons, boas and some colubrids) are non-venomous, while opisthoglyphous (enlarged teeth at the back of the mouth, e.g. cat snakes of the genus *Boiga*), proteroglyphous (fixed-front fanged snakes of the family Elapidae) and solenoglyphous (hinged-front fanged snakes of the family Viperidae) snakes are considered venomous (Kardong, 1982; Persson, 1996). Major families of lytic or apoptotic proteins present in snake venoms include Phospholipase A_2_ (PLA_2_) and three finger toxins (3FT) in elapids, and metalloproteases in vipers (Tasoulis and Isbister, 2017). An array of bacterial species, including *Bacteroides fragilis, Clostridium* spp., *Enterococcus faecalis, Escherichia coli, Morganella morganii*, *Pseudomonas aeruginosa, Serratia marcenscens, Proteus* spp., *Staphylococcus aureus, Staphylococcus epidermidis,* and *Streptococcus* spp., have been identified within the oral cavity of ophidians (Barbosa et al., 2018; Krishnankutty et al., 2019; Lam et al., 2011; Mao et al., 2016; Shek et al., 2009; Theakston et al., 1990). Comparative analyses by Lam et al. (2011) suggest a higher prevalence of pathogenic microorganisms in venomous snakes compared to their non-venomous counterparts, with several microbial species possessing zoonotic potential (Krishnankutty et al., 2019). The oral flora of snakes may contain *Aeromonas* spp., *Bacteroides* spp., *E. coli, Enterococcus* spp.*, Staphylococcus* spp.*, Streptococcus* spp.*, Proteus* spp. and *Pseudomonas* spp., all of which are known to be involved in necrotising fasciitis, a severe condition characterised by the rapid and extensive inflammation and necrosis of subcutaneous fascial tissues (Young et al., 2005).

See Supplementary Table 2 for a breakdown of cases and the species of snake involved and Supplementary Table 8 for a breakdown of pathogens isolated in each case.

#### 3.1.1 Proteroglyphous snakes: cobras, kraits, coral snakes and allies

Tsai et al. (2017) observed that patients presenting with necrotising fasciitis following envenomation by the Chinese Cobra *Naja atra* were frequently infected with *Bacteroides faecilis, E. faecalis,* and *M. morganii.* These bacteria are extensively documented within the oral microbial flora of snakes, and both *B. faecilis and E. faecalis* are known contributors to necrotising fasciitis. These bacteria are inhabitants of the intestinal tract and are commonly associated with faecal matter. As prey tend to defecate when being ingested by snakes (Kerrigan, 1992; Mao et al., 2016), gut bacteria from the prey are passed to the buccal cavity of their predator.

The link between infection and faecal matter-associated bacteria was noted in the case of a child bitten by an unidentified cobra, resulting in swelling, tachycardia and intense pain. The patient visited the hospital two days after the bite, as necrotic fasciitis developed. Debrided tissue revealed *M. morganii* and *E. faecalis*. The patient made a full recovery following antibiotics and skin grafts (Hearn et al., 2015). However, in other instances, amputations may be necessary to prevent further spread of the infection, especially if tissues are deemed to be gangrenous or devitalised. Laohawiriyakamol et al. reported 58 paediatric cases, most requiring debridement following necrosis and *M. morganii* infections and one patient required a toe amputation (Laohawiriyakamol et al., 2011). In more extreme circumstances, entire limbs can require amputation, such as a woman requiring amputation below the hip following an unidentified snakebite (Elserafy, 2023).

**Fig. 1:**
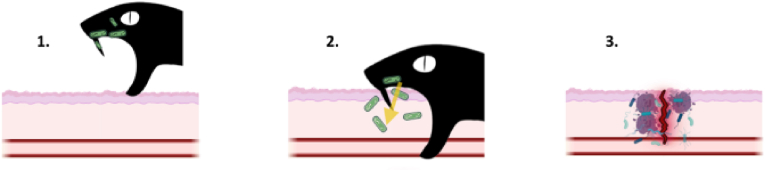
Hypothetical envenoming-to-infection pathway. 1: oral/fang microbes of snake. 2. Microbes vectored into wound during the envenoming process. 3. Resulting infection. Image edited with Biorender.com.

Elapid bites have also been linked to fatal septic shock, as in the case of a 2-year-old child bitten by a spitting cobra *Naja nigricincta nigricincta*. The patient presented with pain, swelling, and skin discoloration. *Proteus vulgaris* was isolated from the necrotic lesion at the bite site. Despite debridement, the infection spread, ultimately leading to the child's death via septic shock (Saaiman and Buys, 2022). Oral swabs from the snake were positive for *P. vulgaris* alongside *E. faecalis, Pseudomonas aeruginosa, M. morganii* and *Salmonella* spp., thus suggesting that *P. vulgaris* was vectored during the bite and that bacterial transmission was responsible, at least in part, for the death of the patient. For hypothetical transmission route, see Fig. 1.

#### 3.1.2 Solenoglyphous snakes: vipers and rattlesnakes

(Paul et al., 2018) report on a male presenting with a viper bite, subsequently treated with polyvalent anti-venom. The patient was admitted with cellulitis and necrotic fasciitis in their lower leg. Despite the amputation of the injured limb, the patient developed septic shock, acute respiratory distress syndrome and multi-organ failure. Bacterial cultures were not performed but incidence of septic shock indicates a widespread and severe infection.

Victims of envenoming by vipers of the genus *Bothrops* have been reported to develop *Aeromonas hydrophila* infections, leading to swelling, pain, and inflammation within a 24-h period, culminating in abscess formation (Jorge et al., 1998). *A. hydrophila* was cultured from the lesions caused by the bite of a suspected South American bushmaster *Lachesis muta* (identification of the snake not confirmed) on the foot of a male victim who presented with swelling, pain, and haematemesis, followed by blistering and progressive necrosis, which required the amputation of the limb (Kallel et al., 2018). While such infections rarely lead to mortality, they may require surgical intervention, including first debridement and grafting, or, if these fail, amputation.

A male and a female bitten by Chinese moccasins *Deinagkistrodon acutus* presented with necrotic fasciitis. *S. aureus, M. morganii, P. aeruginosa, Enterococcus* sp. and *Bacteroides fragilis* were cultured from the wound sites (Cheng et al., 2017). Following a Russel's viper *Daboia russelii* bite, *P. aeruginosa* resistant to fluoroquinolones and cephalosporins was cultured from a necrotic wound in a child (Kaur & Mahajan, 2018). *E. coli* and antibiotic resistant *S. aureus* were isolated from the bite wound of a male presenting pain, swelling, redness and blisters days after being envenomed by a South American rattlesnake *Crotalus durissus collilineatus* (Nishioka et al., 2000).

#### 3.1.3 Opisthoglyphous snakes: venomous colubrids

Opisthoglyphous snakes involved in post-bite infections are less represented in the literature. This paucity is due to the limited number of medically significant opisthoglyphous snakes as most species produce venoms inducing only local effects at the bite site (Weinstein et al., 2013) and sequelae and medical intervention are rare. For instance, no infection was reported by Ballouard et al. (2022) following envenomations in two cases of Montpellier snake *Malpolon monspessulanus* (Ballouard et al., 2022). One of two envenomings by the Plains hognose *Heterodon nasicus* collated from the literature led to cellulitis (Brandehoff et al., 2019; Weinstein and Keyler, 2009). Among eight bites by members of the genus *Boiga*, none progressed to infection (Rathnayaka et al., 2023; Weinstein et al., 2014). Other publications show similar patterns in envenomings by the False Water Cobra *Hydrodynastes gigas* (Keyler et al., 2016) and the South American green racer *Philodryas olfersii latirostris (*Peichoto et al., 2007).

### 3.2 Spiders

Spiders form a hyper-diverse order of arachnids currently comprising 52,813 species (WorldSpiderCatalogue, 2025). While all spiders are venomous, with the exception of members of the family Uloboridae (Bettini and Brignoli, 1978), medically significant spider bites are caused by a very restricted group of species comprising the genera *Atrax*, *Hadronyche*, *Lactrodectus*, *Loxosceles*, *Steatoda* and *Phoneutria* (Isbister and Fan, 2011). Spiders can be broadly grouped depending on the orientation of their chelicerae (vertical in the orthognaths vs lateral in the labidognaths). All medically significant spiders belong to the labidognath spiders, aside from members of the genera *Atrax* and *Hadronyche* (Hedin et al., 2018).

The term “necrotic arachnidism” has been commonly used to explain the occurrence of necrotic wounds following spider bites (Sams et al., 2001). Arachnidism-led dermonecrotic lesions usually involve Sphingomyelinase D (SMD), a non-neurotoxic toxin produced by violin spiders of the genera *Loxosceles* and *Sicarius* (Binford and Wells, 2003), which catalyses the hydrolysis of sphingomyelin, resulting in the breakdown of muscle cell membranes (Ribeiro et al., 2015). However, it has since been demonstrated that most alleged cases of necrotic arachnidism are not supported by sufficient evidence (i.e. the biting incident was not observed, the spider was not recovered) (Vetter et al., 2006). Additionally, investigations into spider venom composition suggest limited cytotoxic and apoptotic toxins in most taxa (Duran et al., 2020; Foradori et al., 2005; Lüddecke et al., 2022; Nentwig and Kuhn-Nentwig, 2012). Spider venom predominantly consists of neurotoxic components.

Studies have identified bacterial species such as *A. hydrophila*, *Bacillus* spp., *Citrobacter amalonaticus, Clostridium perfringens*, *M. morganii, P. aeruginosa, S. aureus*, and *S. epidermidis* on/within the fangs of the Texas tan tarantula *Aphonopelma anax* (McCoy and Clapper, 1979), the Brazilian Brown recluse *Loxosceles intermedia* (Catalán et al., 2010), Western Black widow spider *Latrodectus hesperus* (Ahrens and Crocker, 2011), and the Noble false widow spider *Steatoda nobilis* (Dunbar et al., 2020b). Some spider-associated bacteria exhibit antimicrobial resistance, with instances of *C. perfringens* displaying resistance to tetracyclines and penicillin (Catalán et al., 2010), and various bacterial species showing resistance to erythromycin and nalidixic acid (Dunbar et al., 2020b). Since spider chelicerae are known to harbour bacteria pathogenic to humans, necrotic wounds might be attributable to bacterial infections rather than venom action, or even triggered by cellular apoptosis as a protective mechanism (Dunbar et al., 2019). A study of 761 spider bites in Australia, including those from the Sydney funnel web spider, *Atrax robustus*, found that less than 1 % of bites resulted in infection (Isbister and Gray, 2002). Since *A. robustus* are not known to produce cytotoxic or lytic agents in their venom, these observations suggest that while lytic agents may promote secondary infections, pathogens may be effectively vectored only through the mechanical bite.

See Supplementary Table 3 for a breakdown of cases and spider species involved and see Supplementary Table 9 for a breakdown of pathogens isolated in each case.

#### 3.2.1 Widow spiders

Black widows (genus *Latrodectus*) and False widows (genus *Steatoda*) produce a mostly neurotoxic venom comprising α-latrotoxin, a potent pore-forming toxin targeting neuronal cells (Dunbar et al., 2020a; Orlova et al., 2000; Ushkaryov et al., 2008). α-latrotoxin has a high specific affinity for receptors on vertebrate neuronal cells, causing the cells to release neurotransmitters. A review of 2144 historical case of Red back spiders *Latrodectus hasselti* bites from 1963 to 1976 shows that infections at the bite site were reported as uncommon, with no further details on their occurrence (Sutherland and Trinca, 1978). In a case series involving the Noble false widow spider *Steatoda nobilis* (N = 16), four cases exhibited cellulitis or dermatitis. Most extreme was the case of a woman who experienced three bites in succession from a single specimen, resulting in pain, erythema, and swelling, with subsequent cellulitis, leading to hospitalisation. Blisters and secondary dermatitis ensued, requiring 95 days to reach complete recovery (Dunbar et al., 2022).

#### 3.2.2 Cheiracanthium spp

Yellow sac spiders and other spiders of the genus *Cheiracanthium* are regularly blamed for necrotic arachnidism despite limited evidence suggesting that the genus can produce medically significant skin lesions. In a case series (N = 20) involving patients aged 2–61 years (Vetter et al., 2006), reported symptoms of pain and discomfort but no skin lesions or ulcerations. The authors reviewed 40 additional cases of *Cheiracanthium* bites reported in the literature. A single case, involving a bite by *Cheiracanthium punctorium* reported a necrotic lesion, but no link to infection was made. Ultimately, there is no hard evidence linking *C. punctorium* to medically significant infections.

#### 3.2.3 Loxosceles spp

Violin spiders (Sicariidae: *Loxosceles*) are the spiders most consistently associated with infections in the literature (Broughton, 1996). However, in a review of 120 cases, only 23 % (N = 28) of alleged *Loxosceles* bites were supported by direct evidence; the remaining cases may have resulted from a range of other conditions, as diagnoses were based on non-specific localised symptoms (Lopes et al., 2020; Yigit et al., 2008). All Sicariidae produce SMD as part of their venom, and might therefore be able to produce dermal necrotic lesions following envenoming via sphingomyelin cell lysis.

In a 31-case series of necrotic fasciitis presented by Majeski (2001), two cases were credited to *Loxosceles* spiders. Two patients sought medical attention a week after the envenoming. *Streptococcus* sp.*, E. coli, S. aureus* and *Proteus* sp. were isolated from microbial cultures performed following wound debridement (Majeski, 2001).

Cases where fungal infections are identified as the causative agent following spider envenoming seem unique to *Loxosceles*. For instance, a woman bitten on the leg by a *Loxosceles* sp. initially experienced swelling and pain, progressing to lesions and blistering. Despite treatment with antibacterial vancomycin and meropenem, the wound continued to deteriorate, revealing white mould in the necrotic borders upon debridement. Isolation of *Apophysomyces elegans* prompted treatment with antifungal amphotericin. The patient underwent amputation and ultimately developed sepsis, before passing away (Saravia‐Flores et al., 2010). Tormos et al. (2019) reported on a male bitten by a Chilean recluse *Loxosceles laeta*. The patient presented with pain, swelling, and erythema giving way to blistering. Extensive muscle necrosis reaching the bone required full shoulder disarticulation and amputation. *Saksenaea vasiformes* was isolated from the wound (Tormos et al., 2019). *S. vasiformes* infections are often associated with transmission of soil residing microbes occurring following trauma to the cutaneous layer. Similar trauma would occur during envenomation and piercing via chelicerae. Given proximity of chelicerae to the ground, it is possible that the fungus was vectored in from soil by the fangs during envenoming.

### 3.3 Scorpions

Of the 2363 known species of scorpions spread across 19 families less than 50 species worldwide are thought to be responsible for human fatalities (Lourenço, 2018; Mullen and Sissom, 2019). An estimated 1.2 million stings occur annually, resulting in 2600 deaths, 90 % of which are children under the age of 15 (Chippaux, 2012; Chippaux et al., 2020). Sting incidence varies greatly between regions; the sting incidence in France is approximately five stings per 100,000 people (De Haro et al., 1996), but is about 420 stings per 100,000 people in Tunisia (Njah et al., 2001; Soulaymani Bencheikh et al., 2003).

The venom of most scorpions is neurotoxic, and severe envenoming may lead to autonomic storms (episodes of paroxysmal sympathetic hyperactivity) (Gwee et al., 2002). A restricted number of species have been demonstrated to possess lytic toxins, such as Isalo cytotoxin (IsCT), a pore-forming peptide recovered from the venoms of both *Opisthacanthus madagascarensis* and *Mesobuthus martensii* (Ma et al., 2009). The venom of the Middle eastern Death Stalker *Leiurus quinquestriatus* can induce apoptosis (Omran, 2003).

The microbiota of scorpions remains relatively understudied. Microbes recovered from the stinging apparatus include members of the genera *Staphylococcus, Bacillus, Bordetella, Streptomyces and E. coli* (Shimwell et al., 2023). *S. aureus* isolates from *Hottentotta saulcyi, Androctonus crassicauda, Hemiscorpius lepturus, Compsobuthus matthiesseni* and *Orthochirus iranus* possessed toxin genes and demonstrated varying levels of antimicrobial resistance with two isolates resistant to 10 common antibiotics (Moradikian et al., 2022).

See Supplementary Table 4 for a breakdown of cases and scorpions species involved, and Supplementary Table 10 for a breakdown of pathogens isolated in each case.

Infections following scorpion stings appear to be uncommon, although isolated reports provide evidence that opportunistic post-envenoming infections can occur. In the case of two males stung multiple times by an unidentified scorpion and presenting with skin lesions resulting in infective endocarditis (Wheatley Iii et al., 2005), suggested that scorpions acted as vectors for the *Streptococcus* spp. recovered from the victims' blood samples. In another case, a 19-month-old child succumbed to multi-organ failure following two stings from *Leiurus hebraeus*. *Streptococcus bovis* isolated from post-mortem blood samples (Sofer et al., 1991) was likely vectored into the child from the scorpion sting. In another case, a man was admitted to a Greek hospital 10 days after sustaining a sting to the neck from a presumed *Euscorpius sicanus*. The patient presented with fever, skin necrosis and abscesses, necessitating multiple debridement procedures and ultimately requiring extensive neck reconstruction surgery (Nikolaidou et al., 2022). The reasons for the 10-day delay in treatment were multifactorial but the patient's remote residence and reluctance to seek hospital care were primary factors in the resultant abscesses. In a larger series comprising 1381 cases involving scorpion stings in a hospital in Iran, the incidence rate of infections was 2.8 %, with 29 cases of cellulitis and five cases of necrotizing fasciitis (Alavi and Azarkish, 2011).

In the central region of the Democratic Republic of the Congo, the majority of members of the Lokoko/As’Eto Pygmy tribe stung by scorpions presented with pruritic rashes accompanied by bullae and dermal lesions (Biezakala Mudiandambu et al., 2012) similar to those inflicted by the Middle Eastern species *Hemiscorpius lepturus*. With many victims suffering unsightly scars and lesions, it is possible that stings by unidentified scorpion in the area may lead to infections.

The West Asian Gadim scorpion *Hemiscorpius lepturus* possesses a potent necrotoxic venom containing Heminecrolysin, a 33 kDa protein, similar to the sphingomyelinase found in *Loxosceles* spiders (Borchani et al., 2011; Torabi et al., 2017).

*H. lepturus* is the main contributor to necrosis following scorpion stings and these lesions often heal poorly (Radmanesh, 1998). While Radmanesh's comprehensive study series comprising 489 cases does not specify infections, only 4.5 % of cases lacked skin lesions, and over 40 % presented with necrosis (Radmanesh, 1998). Following a sting by *Hemiscorpius acanthocercus*, a patient rapidly presented with fever, pain and hematuria, followed by necrosis and cellulitis and finally, death (Shahi et al., 2015). Though no cultures were performed, the presence of cellulitis suggests the development of an infection.

Following a scorpion sting to the leg, a boy was infected by the fungus *S. vasiformis*. The infection progressed into necrotising fasciitis requiring debriding (Lechevalier et al., 2008). Pourahmad et al. (2013) reported on a woman presenting with local mucormycosis, including redness and cellulitis, two days following an envenoming from an unidentified scorpion on the thoracic area, later followed by extensive necrosis. The patient recovered after antifungal treatment and reconstructive surgery (Pourahmad et al., 2013).

### 3.4 Hymenoptera

The order Hymenoptera contains approximately 150,000 species of bees, wasps, and ants (Fitzgerald and Flood, 2006). All venomous hymenopterans possess cytolytic compounds in their venom. In bees, melittin hydrolyses cell membranes (Dempsey, 1990) and haemolysis via PLA_2_ can induce myotoxicity and necrosis (Ownby et al., 1997). Wasp venom contains PLA_2_ and hyaluronidase, which metabolise hyaluronic acid in tissues (Fitzgerald and Flood, 2006). Ant venom possess metalloproteases and it has been suggested that some species may produce sphingomyelinases (Touchard et al., 2016).

See Supplementary Table 5 for a breakdown of cases and hymenopteran speies involved. See Supplementary Table 11 for a breakdown of pathogens isolated in each case.

Hymenopteran stings are rarely medically significant outside of allergic reactions and mass envenomings (Vetter et al., 1999) but complications have been reported from single stings. For instance, an individual stung by an unidentified hornet experienced progressive pain and swelling of the head, necessitating hospital admission. Subsequent evaluation revealed an infection of a previous bone graft by *S. aureus*, which required debridement (Maugeri et al., 2017). Ryssel et al. (2007) isolated *S. aureus* and type A *Streptococcus* from an individual presenting with extensive necrosis seven days after a bee sting, necessitating fasciotomy and debridement (Ryssel et al., 2007).

Anderson et al. (1995) presented the case of a child stung on the foot by a bee presenting lesions and eosinophilic fasciitis. The infection was unresponsive to penicillin and cloxacillin. Following debridement and treatment, *P. aeruginosa, S. aureus, E. faecalis* and *Xanthomonas maltophila* were isolated (Anderson et al., 1995).

(Truskinovsky et al., 2001) reported on an individual presenting with intense pain, malaise and purpura and skin lesions two weeks after being stung on the hand by a bee. *Streptococcus pyogenes* was isolated from blood samples. The patient passed away after necrosis (Truskinovsky et al., 2001).

Co-infection of fungus and bacteria can also occur. Following a bee sting to the face, *Candida albicans* and *P. aeruginosa* were found congruently in a patient presenting with facial necrosis and haemolytic *Streptococcus* sp. in blood samples. The patient required intense treatment for seven weeks, including debridement and reconstructive surgery (Richardson and Schmitz, 1997).

As seen above *Staphylococcus* and *Streptococcus* are most commonly associated with bee-associated infections. There are reports of infections by unexpected pathogenic bacteria following bee stings. Liang et al. (2021) reported on a fatal bee sting involving an individual who succumbed to a *Vibrio vulnificus* infection, a highly pathogenic species normally acquired through wound exposure in aquatic environments (Liang et al., 2021). Iron overload is a high risk factor for *V. vulnificus* infection and this patient had a history of hepatitis B cirrhosis. The patient had no recent history of seawater exposure and initial symptoms of the bacterial infection were masked by the presence of the sting, thus delaying accurate diagnosis and appropriate treatment. In another case, a patient was admitted after being infected by *Bartonella henselae*, a microbe typically associated with arthropod (flea) transmission following cat scratches, ten days after an ant sting. The patient recovered after experiencing two pulseless state cardiac arrests (Fritz et al., 2018).

Gkitsaki et al. (2023) isolated *Bacillus* spp., *Staphylococcus capitis, Staphylococcus warnerii, Staphylococcus cohnii* and *Micrococcus* spp from stings of honey bees *Apis mellifera*, and *S. epidermidis, Klebsiella* spp., *E. faecalis,* and *Proteus mirabilis* from the stings of European wasps *Vespula germanica* (Gkitsaki et al., 2023). While most of the bacteria identified were ubiquitous within the environment, some were faecal coliforms. Considering their juxtaposition, faecal matter from the anal region may have contaminated the stinging apparatus.

### 3.5 Centipedes

The class Chilopoda comprises approximately 3500 species of myriapods characterised by their first pair of legs modified into strong venomous claws called forcipules (Dugon, 2017). Centipede venom contains metalloproteases, necrosis-inducing cytokines and myotoxins (Ombati et al., 2018; Undheim and King, 2011; Ward and Rokyta, 2018). As a result, post-envenoming skin lesions are regularly reported (Bush et al., 2001; Logan and Ogden, 1985; O’Donel Alexander and O’Donel Alexander, 1984).

See Supplementary Table 6 for a breakdown of cases and centipede species involved. See Supplementary Table 12 for a breakdown of pathogens isolated in each case.

*S. aureus* was detected in three sting victims who subsequently presented with necrosis and cellulitis (Puzzo et al., 2020; Uzel et al., 2009). Group A *Streptococcus* sp. was isolated in a 78-year-old individual following envenoming by a member of the genus *Scolopendra*, resulting in necrosis, ulcers, and septic shock (Tanaka et al., 2022). Serinken et al. (2005) reported the case of an individual stung by a *Scolopendra mortisans*, presenting with red-blue bullae across the trunk four days post envenoming, later progressing to necrotizing fasciitis and multi-organ failure and death (Serinken et al., 2005). Among 46 cases of centipede sting reported from Hong Kong, one case resulted in necrosis extending down to the subcutaneous layer, necessitating debridement; persistent swelling suggestive of infections was observed in seven additional cases (Fung et al., 2011). A 22 year old bitten by a centipede on the scrotum presented with pain and vomiting (Coffin, 1919). Bullae were observed around the site. Though no infection was noted, the matter of the wound turning slightly necrotic and very exposed would leave it at risk of developing secondary infections.

### 3.6 Aquatic envenomings

Infections associated with aquatic envenomings tend to be caused by aquatic microorganisms (e.g. *Vibrio* and *Photobacteriu*m spp.) of different taxonomic groups than those associated with terrestrial envenomings. Many of these microorganisms, such as *Shewanella putrefaciens* and *Vibrio* spp, are capable of inducing cellulitis and necrosis (Diaz and Lopez, 2015). Aquatic animals, such as fish, can host 10^2^–10^4^ bacteria cm^−2^ on their skin (Austin, 2006). Cnidaria possess microbial communities containing the genera *Mycoplasma*, *Phyllobacterium*, *Ralstonia*, *Shingomonas*, *Tenacibaculum* and *Vibrio* (Peng et al., 2021). Waste disposal from industry and agriculture can introduce further pathogens to the aquatic environment and pharmaceutical waste may contribute to the development of antibiotic resistance in pathogens carried by aquatic animals (de Araújo et al., 2021).

See Supplementary Table 7 for a breakdown of cases and aquatic taxa involved. See Supplementary Table 13 for a breakdown of pathogens isolated in each case.

#### 3.6.1 Cnidaria

While stings by most cnidarians typically result only in pain and erythema stemming from nematocyst envenoming, a small number of species, including box jellyfish (e.g., *Chironex fleckeri*) can induce a severe envenoming syndrome (e.g. Irukandji syndrome). Infective and necrotic jellyfish envenomings represent a minute portion of all jellyfish envenomings. Though global estimates would be difficult to tally, over 23,000 stings were recorded on average each year across study sites located along the Spanish Mediterranean coast; suggesting that over half a million jellyfish stings may occur in Spain annually (Dobson et al., 2024). Jellyfish venom is typically non-necrotic (Brinkman et al., 2012) but PLA_2_, metalloproteases and potassium channel inhibitors have been isolated from the Cannonball jellyfish *Stomolophus meleagris* (Li et al., 2014).

As part of a case series consisting of 57 jellyfish envenoming, Thaikruea and Siriariyaporn (2015) reported three instances of necrosis, two of which tested positive for bacterial infections and required debriding (Thaikruea and Siriariyaporn, 2015). Desax-Willer et al. (2018) and Binnetoglu et al. (2013) have reported two distinct cases involving two children developing skin ulceration and necrosis following jellyfish stings, and required extensive treatment before recovering (Binnetoglu et al., 2013; Desax-Willer et al., 2018).

An issue seen across jellyfish envenoming is the use of first aid measures mostly amounting to home remedies, which amongst other things may delay seeking appropriate medical attention and treatment. The extensive use of inefficient and sometimes harmful folk remedies for jellyfish stings remains a global issue (Headlam, 2020). Shabani et al. (2020) report on a Rhizostomae jellyfish sting treated with lemon juice and baking soda, followed by an onset of soft tissue cellulitis which was treated with antibiotics (Shabani et al., 2020).

Stings from the fire coral *Millepora* sp. may produce rash and erythematous papulae requiring antibiotic treatment (Xu et al., 2020) and an infection by *Photobacterium damselae* was observed following an unidentified coral sting (Badillo et al., 2012). A sting by an unidentified sea anemone led to cellulitis successfully treated with a course of cefuroxime, cloxacillin and metronidazole and ciprofloxacin (Lim and Kumarasinghe, 2007).

#### 3.6.2 Fish

Venomous fish like scorpionfish, catfish and stingrays rely on defensive venom systems located on their dorsal and caudal fin spines (Cameron and Endean, 1966; Fernandez et al., 2011).

##### 3.6.2.1 Scorpionfish

Scorpionfish is a broad term for the groups of fish belonging to Scorpaenidae which include lionfish, stonefish and “true” scorpionfish. Their venom system consists of 13 dorsal spines, three anal spines and two pelvic spines with twinned venom glands (Campos et al., 2017; Halstead et al., 1955; Lennox-Bulow et al., 2023). Though typically neurotoxic, the cytolytic and haemolytic toxin Sp-CTx has been isolated from the Black scorpionfish *Scorpaena plumieri* (Andrich et al., 2010; Shiomi et al., 1989).

Envenomings typically occur when standing on concealed specimens, while maintaining aquaria or fishing. Overall, scorpionfish stings rarely lead to infections (Badillo et al., 2012; Brenneke and Hatz, 2006; Lucerna et al., 2017; Tay et al., 2016). Fifteen cases of envenomings by captive Red lionfish *Pterois volitans* led to two infections (Haddad et al., 2015). In a case series of 23 stings by *S. plumieri* and the Red scorpionfish *Scorpaena brasiliensis,* a single victim presented with a bacterial infection and necrosis (Haddad Jr et al., 2003). *V. vulnificus, Vibrio parahaemolyticus* and *Vibrio cholerae* were recovered from wounds inflicted by the Hollow cheek stonefish *Synanceia horrida* and the Reef stonefish *Synanceia verrucosa* (Tang et al., 2006). *V. parahaemolyticus* and *V. cholerae* were found in a patient who required debriding following an envenoming from the Reef stonefish *S. verrucosa* (Issack et al., 2008).

##### 3.6.2.2 Catfish

Catfish form a diverse group encompassing over 3000 species of which 1250–1625 are presumed venomous. Their venom systems consist of glands associated with spines along the dorsal and pectoral fins. Once threatened, the spines can inject neurotoxic and haemolytic compounds leading to pain and respiratory distress (Wright, 2009).

*P. damselae,* a relative of the *Vibrio* genus, has been involved in the fatal case a man who contracted the infection from a catfish barb sting (Clarridge and Zighelboim-Daum, 1985; Hargreaves and Lucey, 1990). *V. vulnificus* and *V. cholerae* were isolated in two other cases (Baack et al., 1991; Bonner et al., 1983), one of which required the amputation of two fingers. Baack et al. (1991) reported co-infections from catfish stings, involving *Edwardsiella tarda, E. coli, Klebsiella pneumoniae* and *E. tarda. A. hydrophila, Citrobacter freundii* and *Fusobacterium mortiferum* were all involved in the non-lethal infection of a man following a catfish sting (Hargreaves and Lucey, 1990).

##### 3.6.2.3 Stingrays

Stingrays rely on their barbed caudal fin spine to inject venom. Most envenomings occur when the victim accidently steps on the stingray, generally leading to envenomings to the lower foot (Clark et al., 2007). In a case series following 22 stingray stings, *S. aureus* and *V. vulnificus* were involved in at least four out of eight cases resulted in infections (Cevik et al., 2022)*.* Sepsis due to *Streptococcus mitis, Eikenella corrodens, Actinomyces odontolyticus, E. faecalis* and *Staphylococcus agalactiae* have been reported (Bendt and Auerbach, 1991; Hønge et al., 2018), as well as severe infections by other common aquatic bacteria such as *P. damselae* (Barber and Swygert, 2000), *V. parahaemolyticus* (Bonner et al., 1983) and *Vibrio alginolyticus* (Ho et al., 1998). In a 119-case series, less than 7 % of cases presented with infections, suggesting that infection is not a common occurrence in stingray stings (Clark et al., 2007).

##### 3.6.2.4 Octopus

Members of the family Octopodidae produce primarily neurotoxic toxins, including hapalotoxin and tetrodotoxin (TTX) (Hodgson, 1997; Undheim et al., 2010). The biosynthetic origins of the highly potent TTX are uncertain, with both eukaryotic and prokaryotic sources being proposed. It has been suggested that TTX-producing bacteria could be transferred during the envenoming event. (Hwang et al., 1989). *Pseudomonas oryzihabitans* was isolated from a necrotic ulcer resulting from a bite by the Common octopus *Octopus vulgaris* (Aigner et al., 2011) and *V. alginolyticus* was recovered from the ulcerative wound caused by the bite of an unidentified octopus (Campanelli et al., 2008). It remains unclear if the bacteria involved in these cases either originate from the venom system and are vectored into the wound during envenomings or from the environment.

## 4 Discussion

**Fig. 2:**
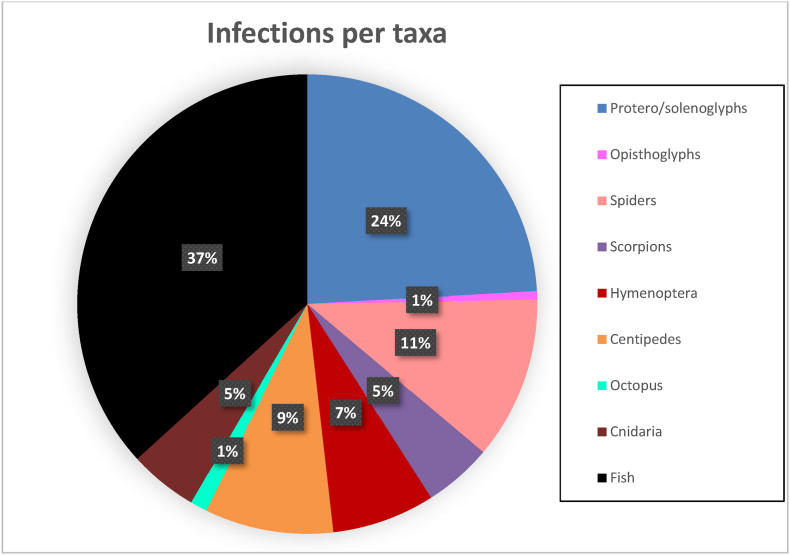
Breakdown of each taxa implicated during an envenoming-led infection (n = 166) from the literature collated for this manuscript.

**Fig. 3:**
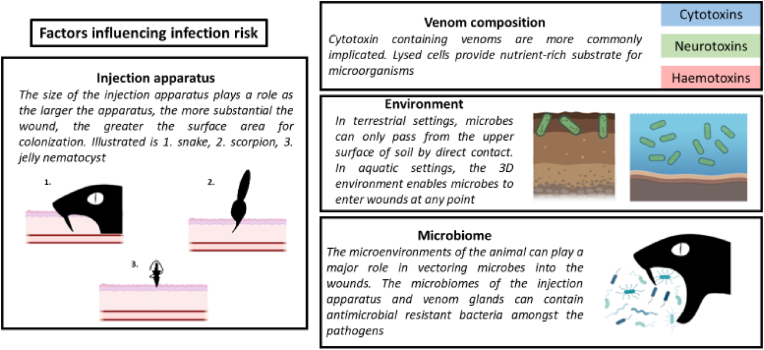
Factors that can contribute to infection risk following envenoming. These include the size of the injection apparatus, venom composition, nature of the environment in which envenoming took place and the particular microbiome associated with the implicated animal. Figure edited using Biorender.com.

Within the literature collated for this review(N = 166), 24 % (N = 40) of envenoming-led infections involved snakes, 37 % (N = 61) involved fish and 11 % (N = 19) involved spiders (Fig. 2). There are several potential factors that can heighten the risk of infection following envenoming (Fig. 3). These factors encompass the biology of these animals from the composition of the venom, to the interior and external microenvironments for microbes to live, and the nature of the animal's delivery system for venom.

From the modified spines and barbs of scorpion fish, catfish and rays to the hollow, hinged fangs of solenoglyphous snakes, the morphology, physiology and evolutionary trajectory of venom systems differ greatly across venomous taxa. Yet these structures always rely on a piercing device to break the integrity of the outer epidermal layer of their target to inject venom. While larger injecting apparatus may produce more substantial and deeper wounds for microbes to settle in, the size and depth of a wound alone do not always seem to correlate with higher risk of infections. For example, bites from comparatively small *Loxosceles* spiders are much more commonly associated with infection compared to those delivered by large tarantulas.

The insertion sub-dermally of any sharp object carries risk of infection. Necrosis and apoptosis of cells following envenomation, especially by organisms producing lytic venoms, may heighten the risk of infection by providing an ideal medium for pathogens to develop.

In this context, venom composition appears to be the main contributor in the envenoming-to-infection pathway. Cytotoxins may cause enough cell lysis for dead tissues to act as a nutrient-rich substrate that promotes microbial growth. The complex cocktails of proteolytic enzymes, peptide hyaluronidases and phospholipases of vipers (Reading, 1996), or cytotoxins, phospholipases and metalloproteinases typical of elapids (Palasuberniam et al., 2021) are much more conducive to infections compared to non-venomous snakes. Similarly, the low rate of infections following bites by opisthoglyphous snakes may be explained by the lack of anterior hollow fangs to efficiently inject venom, despite the presence of metalloproteases and phospholipases in their venom (Koua et al., 2022; Mackessy et al., 2020).

The literature dedicated to envenomation-led infections rarely consider how micro-organisms colonise the wound, i.e., if these are opportunistic commensals of the victim, drawn from the environment in which the venomous organism lives in, or if colonies are specific to certain venomous organisms. In aquatic envenomings, bacteria are likely vectored into the wound from the surrounding medium (Fig. 2), where they are present in high concentration. For example, *Prochlorococcus* spp. can number 4.3-5.12x10^4^ cell ml^−1^ (Ferreira et al., 2022) in upper sea water. This is further supported by reports of envenoming-led *Vibrio* spp. and *Photobacterium* spp. infections (Campanelli et al., 2008; Cevik et al., 2022; Coffey et al., 1986), both of which are free-living in the water column.

In terrestrial organisms, microorganisms may be acquired through contact with prey and the habitat substrate. Many of the bacteria found in the oral cavity of terrestrial venomous snakes are associated with gastrointestinal systems (Kerrigan, 1992; Mao et al., 2016; Tsai et al., 2017). These oral bacteria are probably acquired from prey defecation following predation. Microbial cultures of spider chelicerae reveal antimicrobial resistant bacteria associated with the environment (Dunbar et al., 2020b; Esmaeilishirazifard et al., 2022) though some gastrointestinal species, such as *E. coli,* have also been recovered (Majeski, 2001). Venomous taxa with non-oral associated envenomation apparatus harbour less gastrointestinal species and more environmental species (Shimwell et al., 2023).

Overall, infections may occur irrespective of medical intervention timing. Early symptoms of infection such as erythema, pain and swelling may be camouflaged by the primary symptoms of envenomings and therefore go unnoticed until the infection sets in. This can lead to delayed diagnosis and subsequent treatment. While the reluctance to prematurely administer antibiotics may be warranted, medical practitioners should consider infection as a risk associated with envenomings.

Our knowledge of specific associations between microorganisms and venom systems remains limited, and further work is required to understand the relationship between venomous organisms and the potential harmful microbiome they harbour. Increasing our understanding of these topics is imperative for the future so that appropriate judgements can be made on post-envenoming care, such as selection of antibiotic treatment. Greater understanding and recognition of infection as a potential sequelum will enable swift effective action to be taken following envenoming in order to minimise and prevent mortality and morbidity.

## CRediT authorship contribution statement

**Dayle Leonard:** Writing – original draft, Visualization, Resources, Methodology, Investigation, Data curation. **Aoife Boyd:** Writing – review & editing, Visualization, Supervision, Conceptualization. **Michel M. Dugon:** Writing – review & editing, Supervision, Project administration, Methodology, Conceptualization.

## Key points

• Infection is a serious risk following envenoming that can increase mortality and life altering morbidity
• Pathogen species at envenomation sites derive from a variety of sources depending on nature of the incident
• The size of the venom delivery apparatus influences the risk of infection with larger venom systems of snakes and fish resulting in more substantial wounds for colonisation by micro-organisms
• Venom containing cytotoxic compounds are more often implicated in infection

## Ethical statement

This manuscript is based on publicly available, previously published peer-reviewed articles. This manuscript does not contain any sensitive or personal information that may allow the identification of patients.

## Declaration of competing interest

The authors declare that they have no known competing financial interests or personal relationships that could have appeared to influence the work reported in this paper.

## Data Availability

Data was gathered from published literature in public databases.
