# Supplementary material for: Adding insult to injury: A review of infections following envenomings
Source: Toxicon X. 2025 Jun 23;27:100230. doi: 10.1016/j.toxcx.2025.100230 (PMC12268858; doi:10.1016/j.toxcx.2025.100230)
Supplement: Multimedia component 1 [file mmc1.docx]

**Supplementary table** **section**

**Supplementary table** ***1:*** *A list of the search terms used in the literature collating process. Presence of “-“ denotes lack of alternate term used.* NCBI Pubmed and Google Scholar databases were utilised to attain hits for search criteria. *Retained means it passed review and was included in this manuscript*

| Scientific name | Vernacular name | Returned hits from databases | # of studies selected | # of studies retained |
| --- | --- | --- | --- | --- |
| - | Snake | 61,900 | 20 | 8 |
| - | Cobra | 2,110 | 27 | 9 |
| - | Viper | 4,470 | 23 | 13 |
| - | Elapid | 4,890 | 3 | 0 |
| - | Colubrid | 2,660 | 19 | 11 |
| *Crotalus sp* | Rattlesnake | 2,560 | 16 | 3 |
| *Daboia russeli* | Russel's viper | 625 | 31 | 2 |
| *Bothrops sp* | Pit viper | 536 | 18 | 4 |
| *Protobothrops sp* | Asian pit viper | 860 | 14 | 6 |
| *Ophiophagus hannah* | King Cobra | 1,590 | 10 | 1 |
| *Heterodon sp* | Hognose snake | 831 | 7 | 2 |
| *Heterodon nasicus* | Western hognose | 229 | 7 | 2 |
| *Boiga sp* | Cat snake | 1,230 | 6 | 2 |
| *Dispholidus typus* | Boomslang | 1,050 | 6 | 1 |
| *Hydrodynastes gigas* | False water cobra | 106 | 2 | 2 |
| *Lachesis muta* | South American Bushmaster | 1,670 | 7 | 1 |
| *Arachnid* | Spider | 81,800 | 45 | 3 |
| Theraphosid | Tarantula | 7,350 | 11 | 1 |
| *Latrodectus sp* | Black widow spider | 3,520 | 27 | 2 |
| *Steatoda nobilis* | False widow spider | 141 | 2 | 1 |
| *Loxosceles sp* | Brown recluse/violin spider | 3,580 | 17 | 12 |
| *Phoneutria sp* | Brazilian wandering spider | 1,010 | 6 | 2 |
| *Cheiracanthium* | Yellow sac spider | 410 | 8 | 3 |
| *Sicarius* | - | 392 | 1 | 0 |
| - | Scorpion | 30,500 | 21 | 11 |
| *Hemiscorpius lepturus* | West Gadim scorpion | 774 | 5 | 3 |
| *Leiurus quinquestriatus* | Deathstalker scorpion | 1,200 | 6 | 3 |
| *Androctonus sp* | Fattail scorpion | 2,110 | 5 | 1 |
| *Hottentotta sp* | - | 551 | 4 | 1 |
| *Buthus sp* | - | 1,950 | 5 | 1 |
| Hexapod | Insect | 617 | 1 | 1 |
| *-* | Ant | 127,000 | 6 | 1 |
| *-* | Wasp | 33,900 | 9 | 4 |
| *Vespa sp* | Hornet | 10,200 | 8 | 3 |
| *Apidae* | Bee | 83,800 | 16 | 5 |
| *Apis mellifera* | Honeybee | 11,300 | 7 | 1 |
| *Solenopsis sp* | Fire ant | 4,540 | 3 | 1 |
| Hymenoptera | - | 27,300 | 3 | 0 |
| *-* | Centipede | 7,290 | 13 | 5 |
| *Scolopendra sp* | - | 1,130 | 11 | 6 |
| *Cnidaria* | Jellyfish | 9,960 | 28 | 9 |
| *Millepora sp* | Firecoral | 506 | 6 | 3 |
| *-* | Coral | 24,500 | 7 | 1 |
| *-* | Sea anenome | 5,530 | 7 | 1 |
| *Aurelia aurita* | Moon jelly | 769 | 6 | 1 |
| *Physalia sp* | Portuguese Man of war | 1,400 | 5 | 1 |
| *-* | Sea urchin | 2,680 | 8 | 2 |
| *-* | Octopus | 17,500 | 5 | 4 |
| *Hapalochlaena sp* | Blue ring octopus | 321 | 3 | 2 |
| *Dasyatis sp* | - | 1,910 | 1 | 1 |
| *-* | Stingray | 3,960 | 28 | 13 |
| *-* | Catfish | 6,570 | 19 | 15 |
| *Scorpaenidae* | Scorpionfish | 636 | 11 | 7 |
| *Pterois sp* | Lionfish | 671 | 10 | 5 |
| *Synanceia sp* | Stonefish | 403 | 8 | 2 |
|  | Totals | 606,998 | 608 | 205 |

**Supplementary table *2:*** *Data table of information taken from each snake envenoming case involved in the review*

| Sex | Location | Infection | Species | Group | Paper |
| --- | --- | --- | --- | --- | --- |
| f | Foot | No | *Daboia russelii* | viper | Ghosh, R., et al. (2022). |
| m | Hand | Yes | rattlesnake | rattlesnake | Gelman, D., et al. (2022 |
| m | leg | Yes | cobra | cobra |  |
| m | leg | yes | cobra | cobra |  |
| m | hand | Yes | cobra | cobra |  |
| m | leg | Yes | cobra | cobra |  |
| f | leg | Yes | cobra | cobra |  |
| m | hand | Yes | cobra | cobra |  |
| m | leg | Yes | cobra | cobra |  |
| f | leg | Yes | cobra | cobra |  |
| f | leg | Yes | cobra | cobra |  |
| m | hand | Yes | cobra | cobra |  |
| m | hand | Yes | cobra | cobra |  |
| m | arm | No | cobra | cobra |  |
| f | hand | No | cobra | cobra |  |
| f | hand | No | cobra | cobra |  |
| f | hand | No | *Protobothrops mucrosquamatus* | viper |  |
| f | leg | Yes | cobra | cobra | Tsai, Y.-H., et al. (2017). |
| m | hand | Yes | viper | viper | Nadiyah, A., et al. (2015). |
| f | leg | Yes | snake | snake | Elserafy, O. S. I. S. (2023). |
| f | foot | Yes | snake | snake | Cheong, C. Y., et al. (2010). |
| m | foot | Yes | *Bothros* *moojeni* | viper |  |
| m | hand | Yes | *Bothrops* sp | viper |  |
| m | foot | yes | *Bothrops* sp | viper | Jorge, M. T., et al. (1998). |
| m | leg | no | *Deinagkistrodon* sp | viper | Xu, A., et al. (2016). |
| m | foot | yes | *Crotalus durissus collilineatus* | rattlesnake | Nishioka, S. d. A., et al. (2000). |
| m | hand | no | *Malpolon monspessulanus* | colubrid |  |
| m | hand | no | *Malpolon monspessulanus* | colubrid | Ballouard, J.-M., et al. (2022). |
| m | face | yes | *Naja nigricinta nigricinta* | cobra | Saaiman, E. L. and P. J. Buys (2022). |
| m | arm | yes | snake | snake | Sadeghi, M., et al. (2021). |
| f | arm | yes | snake | snake | Banda, C. H. and C. N’gambi (2019). |
| m | hand | yes | *Deinagkistrodon acutus* | viper |  |
| f | foot | yes | *Deinagkistrodon acutus* | viper |  |
| f | hand | yes | *Deinagkistrodon acutus* | viper | Cheng, C.-L., et al. (2017). |
| m | foot | yes | viper | viper | Kallel, H., et al. (2018). |
| f | hand | no | *Thamnodynastes pallidus* | colubrid | Araújo, P. F. d., et al. (2018). |
| f | hand | yes | viper | viper | Balaji, G., et al. (2015). |
| m | hand | no | *Erthyrolampus aesculapii* | colubrid | Menegucci, R. C., et al. (2019). |
| m | leg | yes | *Ophiophagus hannah* | cobra |  |
| m | hand | no | *Ophiophagus hannah* | cobra | Le, H. Q., et al. (2021). |
| f | foot | yes | *Daboia russelii* | viper | Kaur, H. and G. Mahajan (2018). |
| m | leg | yes | snake | snake | Chidambaram, M., et al. (2022). |
| m | arm | yes | cobra | cobra | Hearn, P., et al. (2015). |
| f | arm | yes | *Heterodon nasicus* | colubrid | Weinstein, S. A. and D. E. Keyler (2009). |
| f | hand | no | *Heterodon nasicus* | colubrid | Brandehoff, N., et al. (2019). |
| m | leg | yes | viper | viper | Paul, R., et al. (2018). |
| m | hand | no | *Lachesis muta muta* | viper | Tanus Jorge, M., et al. (1997). |
| m | genitals | no | *Crotalus atrax* | rattlesnake | Crane, D. B. and J. S. Irwin (1985). |
| f | leg | no | viper | viper | Bucaretchi, F., et al. (2019). |
| m | hand | no | *Boiga ceylonensis* | colubrid |  |
| m | foot | no | *Boiga ceylonensis* | colubrid |  |
| m | foot | no | *Boiga forsteni* | colubrid |  |
| f | hand | no | *Boiga forsteni* | colubrid |  |
| m | arm | no | *Boiga ceylonensis* | colubrid |  |
| m | arm | no | *Boiga ranawanei* | colubrid |  |
| m | hand | no | *Boiga ranawanei* | colubrid | Rathnayaka, R. M. M. K. N., et al. (2023). |
| f | hand | no | *Boiga dendrophila* | colubrid |  |
| m | hand | no | *Rhamphiophis oxyrhynchus* | colubrid |  |
| m | hand | no | *Leptodeira frenata* | colubrid | Weinstein, S. A., et al. (2014). |
| m | trunk | no | *Dispholidus typus* | colubrid | Geddes, J. and J. E. P. Thomas (1985). |
| various | various | yes x 33 | snake | snake |  |
| various | various | no x 10 | snake | snake | Garg, A., et al. (2009). |
|  |  | no x 28 | *Caloselasma rhodostoma* | viper |  |
| various | various | no x 11 | cobra | cobra |  |
| various | various | yes x 10 | cobra | cobra |  |
| various | various | no x 9 | snake | snake | Laohawiriyakamol, S., et al. (2011). |
| m | hand | no | *Philodryas olfersii latirostris* | colubrid | Peichoto, M. E., et al. (2007). |
| m | hand | no | *Hydrodynastes gigas* | colubrid |  |
| m | arm | no | *Philodryas olfersii* | colubrid |  |
| m | hand | no | *Hydrodynastes gigas* | colubrid | Keyler, D. E., et al. (2016). |
| m | foot | yes | *Naja atra* | cobra | Huang, W.-H., et al. (2021). |
| f | foot | yes | *Naja atra* | cobra | Liu, P.-Y., et al. (2014). |
| m | hand | no | *Langaha madagascarensis* | colubrid | D'Cruze, N. C. (2008). |
| m | hand | no | *Ithycyphus miniatus* | colubrid |  |
| m | hand | no | *Ithycyphus miniatus* | colubrid | Mori, A. and T. Mizuta (2006). |

**Supplementary table *3:*** *Data table of information taken from each spider envenoming case involved in the review*

| Age | Sex | Location | Infection | Animal | Paper |
| --- | --- | --- | --- | --- | --- |
| 37 | f | arm | no |  |  |
|  | f | hand | no |  |  |
| 35 | f | body | no |  |  |
| 26 | f | face | no |  |  |
| 52 | f | arm | yes |  |  |
| 44 | f | leg | no |  |  |
| 56 | f | body | no |  |  |
| 56 | m | body | no |  |  |
| 47 | m | arm | no |  |  |
| 46 | f | foot | no |  |  |
| 34 | f | leg | no |  |  |
| 5 | m | body | no |  |  |
| 33 | f | arm | no |  |  |
| 7 | m | hand | yes |  |  |
| 43 | f | leg | yes |  |  |
| 38 | f | arm | no | *Steatoda nobilis* | Dunbar, J. P., et al. (2022) |
| 48 | m | arm | yes |  |  |
| 52 | m | arm | no |  |  |
| 37 | m | arm | no |  |  |
| 54 | f | arm | no |  |  |
| 33 | m | arm | no |  | Dunbar, J. P., et al. (2018) |
| 71 | m | arm | yes | *Loxosceles laeta* | Tormos, E. F., et al. (2019) |
| 35 | m | hand | no | *Psalmopoeus irminina* | Simon, M. and C. Hoyte (2023) |
| 65 | f | hand | no | *Loxosceles rufenscens?* | Pezzi, M., et al. (2016) |
| 33 | f | leg | yes | *Loxosceles rufenscens?* | Coutinho, I., et al. (2014) |
| 27 | m | arm | yes | *Loxosceles laeta* | Harz-Fresno, I., et al. (2015) |
| 14 | f | arm | no | *Loxosceles reclusa* |  |
| 67 | m | extremity | yes |  |  |
| 73 | f | extremity | yes | *Loxosceles sp* | Majeski, J. (2001) |
| 19 | m | genitals | no | *Loxosceles sp.* | Broughton, G. (1996) |
| 27 | m | leg | no | *Loxosceles reclusa* | King, L. E., Jr. and R. S. Rees (1983) |
| 30 | f | leg | yes | *Loxosceles sp.* | Saravia‐Flores, M., et al. (2010) |
| 30 | f | leg | yes | spider | Özyurt, S., et al. (2013) |
| 28 | f | foot | no | *Latrodectus hasselti* | Sutherland, S. K. and J. C. Trinca (1978) |
| 30 | m | hand | no |  |  |
| 7 | f | hand | no | *Cheiracanthium punctorium* |  |
|  |  | hand | no |  |  |
|  |  | hand | no |  |  |
|  |  | hand | no | *Cheiracanthium punctorium* | Varl, T., et al. (2017) |
| 31 | m | foot | no | *Cheiracanthium sp* | Fasan, M., et al. (2008) |
| 42 | f | hand | no | *Phoneutria depilata* | Miranda, R. J., et al. (2022) |
| 52 | m | neck | no | *Phoneutria nigriventer* | Bucaretchi, F., et al. (2008) |
| Various | Various | Various | no x754 | spider |  |
| Various | Various | Various | yes x7 | spider | Isbister, G. K. and M. R. Gray (2002) |
| 20s | f | leg | no | spider | Cachia, M., et al. (2016) |
| 46 | m | foot | no | *Loxosceles reclusa* | Delasotta, L. A., et al. (2014) |
| 34 | f | foot | no | *Latrodectus hasselti* | Weinstein, S. A. (2024) |
| Various | Various | Various | no x 20 | *Cheiracanthium spp* |  |
| Various | Various | Various | no x 40 | *Cheiracanthium spp* | Vetter, R. S., et al. (2006) |
| 24 | f | arm | no | *Loxosceles reclusa* | Rodríguez-Roiz, J. M., et al. (2017) |
| 55 | f | face | no |  |  |
| 54 | f | face | no |  |  |
| 39 | f | face | no | *Loxosceles reclusa* | Leach, J., et al. (2004) |
| 44 | f | leg |  |  |  |
| 31 | f | leg |  |  |  |
| 24 | m | leg |  |  |  |
| 27 | f | leg |  |  |  |
| 45 | f | leg |  |  |  |
| 42 | f | hand |  |  |  |
| 44 | m | arm |  |  |  |
| 31 | m | hand |  |  |  |
| 54 | m | leg |  |  |  |
| 18 | f | leg |  |  |  |
| 15 | f | arm |  |  |  |
| 28 | f | arm |  |  |  |
| 48 | f | face |  |  |  |
| 45 | f | leg |  |  |  |
| 19 | f | leg |  |  |  |
| 42 | m | leg |  |  |  |
| 18 | m | arm |  |  |  |
| 30 | m | foot |  |  |  |
| 18 | m | arm | no | *Loxosceles reclusa* | Sams, H. H., et al. (2001) |
| 25 | f | foot | no | spider | Yigit, N., et al. (2008) |

**Supplementary table** ***4:*** *Data table of information taken from each scorpion envenoming case involved in the review*

| Age | Sex | Location | Infection | Animal | Paper |
| --- | --- | --- | --- | --- | --- |
| 55 | m | N/a | yes | Scorpion |  |
| 34 | m | N/a | yes | Scorpion | Wheatley Iii, G. H., et al. (2005) |
| 30 | m | foot | no | *Palamnaeus bengalensis* | Shah, P. K. D., et al. (1989) |
| 9 | m | foot | yes | *Hottentota jayakari salei* | Suresh, S. S., et al. (2014) |
| 60 | m | neck | yes | *Euscorpius sicanus* | Nikolaidou, E., et al. (2022) |
| 17 | f | hand | no | scorpion | Ansari, M. Y. (1948) |
| 28 | f | arm | N/a | scorpion | Chadha, J. S. and A. Leviav (1979) |
| 7 | f | leg | no | scorpion | Valavi, E. and M. J. A. Ansari (2008) |
| 7m-2yo | various | various | no x 8 | *Leiurus hebraeus* |  |
|  |  |  | no | *Buthotus judaicus* |  |
| 19m | f | n/a | yes | *Leiurus hebraeus* | Sofer, S., et al. (1996) |
| 14 | m | leg | yes | Scorpion | Lechevalier, P., et al. (2008) |
| 55 | f | body | yes | Scorpion | Pourahmad, M., et al. (2013) |
| 12 | m | foot | no | *Odontobuthus doriae* | Razi, E. and E. Malekanrad (2008) |
| 55 | m | n/a | no | Scorpion | El Fortia, M., et al. (2005) |
| various | various | various | yes x 34 | Scorpion |  |
| various | various | various | no x 1347 | Scorpion | Alavi, S. M. and A. R. Azarkish (2011) |
| various | various | various | no x 14 | *Leiurus quinquestriatus* |  |
| various | various | various | no | *Buthotus judaicus* | Sofer, S., et al. (1991) |
| 23 | f | body | no | *Hemiscorpius lepturus* |  |
| 17 | f | leg | no | *Hemiscorpius lepturus* |  |
| 22 | m | body | no | *Hemiscorpius lepturus* |  |
| 17 | m | leg | no | *Hemiscorpius lepturus* | Shayesteh, A. A., et al. (2011) |
| 15 | m | arm | yes | *Hemiscorpius acanthocercus* | Shahi, M., et al. (2015) |
| 12 | f | various | no | *Hemiscorpius acanthocercus* | Shahi, M., et al. (2020) |

**Supplementary table** ***5:*** *Data table of information taken from each hymenopteran envenoming case involved*

| Age | Sex | Location | Infection | Animal | Paper |
| --- | --- | --- | --- | --- | --- |
| 95 | m | face | no | Wasp | Nakatani, Y., et al. (2013) |
| 24 | m | Multi | no | Hornet | Bhattarai, U., et al. (2023) |
| 29 | m | N/a | no | unknown | Freeman, T. M. (2004) |
| 71 | m | Hand | yes | bee | Truskinovsky, A. M., et al. (2001) |
| 61 | m | face | yes | bee | Richardson, D. and J. P. Schmitz (1997) |
| 4 | f | foot | yes | bee | Anderson, C. R., et al. (1995) |
| 40 | f | hand | no | wasp | Kumar, V., et al. (2013) |
| 53 | m | hand | yes | bee | Liang, J.-H., et al. (2021) |
| 61 | m | multi | no | wasp | Zhang, R., et al. (2001) |
| 56 | m | body | yes | bee | Ryssel, H., et al. (2007) |
| 48 | m | head | yes | hornet | Maugeri, R., et al. (2017) |
| 8 | m | leg | yes | yellowjacket | Can, C., et al. (2021) |
| 2 | f | Multi | no | hornet | Akinsola, B. and D. Young (2021) |
| 67 | m | hand | yes | Hornet |  |
| 66 | m | leg | yes | yellowjacket | Koçer, U., et al. (2003) |
| 79 | f | foot | yes | Solenopsis fire ant | Jyothi, R. M., et al. (2008) |
| 56 | m | leg | yes | ant | Fritz, A., et al. (2018) |
| 37 | m | hand | yes | Apis mellifera | Liew, K. C., et al. (2022) |

**Supplementary table** ***6:*** *Data table of information taken from each centipede envenoming case involved in the review*

| Age | Sex | Location | Infection | Animal | Paper |
| --- | --- | --- | --- | --- | --- |
| 60 | m | foot | no | Centipede | Ozsarac, M., et al. (2004) |
| 30 | m | leg | no | *Scolopendra sp* |  |
| 36 | m | hand | no | *Scolopendra heros* |  |
| 37 | m | hand | no | *Scolopendra subspinipes* |  |
| 37 | m | hand | no | *Scolopendra subspinipes* |  |
| 37 | m | hand | no | *Scolopendra subspinipes* |  |
| 37 | m | hand | no | *Scolopendra subspinipes* | Bush, S. P., et al. (2001) |
| various | various | various | yes x 7 |  |  |
| various | various | various | no x38 |  |  |
| 66 | f | hand | yes | Centipede | Fung, H. T., et al. (2011) |
| 16 | m | hand | yes | Centipede | Uzel, A. P., et al. (2009) |
| 46 | M | Body | yes | *Scolopendra mortisans* | Serinken, M., et al. (2005) |
| 63 | m | hand | yes | *Scolopendra subspinipes* | Puzzo, A., et al. (2020) |
| 79 | m | hand | no | *Scolopendra heros* | Essler, S. E., et al. (2017) |
| 78 | m | body | yes | *Scolopendra sp.* | Tanaka, Y., et al. (2022) |
| 22 | m | genitals | no | Centipede | Coffin, S. (1919) |
| 44 | f | foot | no | *Scolopendra heros* | Logan, J. L. and D. A. Ogden (1985) |
| 68 | f | hand | no | Centipede | Friedman, I. S., et al. (1998) |

**Supplementary table** ***7:*** *Data table of information taken from each aquatic animal envenoming case involved in the review*

| Age | Sex | Location | Infection | Animal | Paper |
| --- | --- | --- | --- | --- | --- |
| 45 | m | foot | yes | Stingray |  |
| 32 | m | leg | yes | Sea anemone |  |
| 27 | m | arm | yes | Jellyfish | Lim, Y. L. and S. P. W. Kumarasinghe (2007) |
| 7 | f | leg | no | *Millepora sp.* (Fire coral) | Xu, D., et al. (2020) |
| 27 | f | leg | no | Brain coral | Dyson, M. E. and A. Kimyai-Asadi (2022) |
| 43 | m | leg | yes | Stingray | Barber, G. R. and J. S. Swygert (2000) |
| 52 | f | hand | no | *Millepora sp*. (Fire coral) | Moats, W. E. (1992) |
| 30 | f | leg | no | *Millepora complanata* | Kropp, L. M., et al. (2018) |
| 4 | f | leg | yes | Jellyfish | Desax-Willer, D., et al. (2018) |
| 4 | m | hand | yes | Jellyfish | Binnetoglu, F. K., et al. (2013) |
| 27 | f | arm | yes | Jellyfish | Shabani, M., et al. (2020) |
| various | various | various | no x 54 | box jellyfish |  |
| 21 | m | leg | no | box jellyfish |  |
| 25 | f | multi | yes | box jellyfish |  |
| 33 | f | leg | yes | box jellyfish | Thaikruea, L. and P. Siriariyaporn (2015) |
| 38 | m | leg + foot | no | Jellyfish + sea urchin | Lee, N.-S., et al. (2001) |
| 32 | m | arm | no | *Rhopilema nomadica* |  |
| 37 | m | face | no | *Rhopilema nomadica* | Silfen, R., et al. (2003) |
| 49 | f | foot | no | *Physalia sp* | Auerbach, P. S. and J. T. Hays (1987) |
| 54 | m | face | no | *Aurelia aurita* |  |
| 49 | m | face | no | *Aurelia aurita* | Mao, C., et al. (2016) |
| 23 | m | hand | no | Sea urchin |  |
| 31 | f | hand | no | Sea urchin |  |
| 43 | m | hand | no | Sea urchin |  |
| 37 | m | hand | no | Sea urchin |  |
| 51 | f | hand | no | Sea urchin | Wada, T., et al. (2008) |
| 49 | m | hand | no | *Haliotis mariae* | Al-Kathiri, L., et al. (2019) |
| 51 | m | hand | yes | Octopus | Campanelli, A., et al. (2008) |
| 9 | m | arm | yes | *Octopus vulgaris* | Aigner, B. A., et al. (2011) |
| 53 | f | hand | no | *Octopus vulgaris* | Haddad Jr, V. and C. A. de Magalhães (2014) |
| 31 | f | hand | no | *Octopus rubescens* | Douglas-Vail, M. B., et al. (2023) |
| 35 | m | leg | yes | Stingray | Jiang, T., et al. (2020) |
| 18 | m | trunk | no | *Dasyatis sephen* |  |
| 30 | m | foot | no | *Dasyatis kuhlii* | Barss, P. (1984) |
| 25 | m | arm | no | *Rhopilema hispidum* |  |
| 25 | m | arm | no | *Rhopilema esculentum* |  |
| 25 | m | arm | no | *Nemopilema nomurai* | Kawahara, M., et al. (2006) |
| 5 | m | leg | yes | freshwater catfish |  |
| 51 | m | hand | yes | freshwater catfish | Murphey, D. K., et al. (1992) |
| 68 | m | hand | yes | catfish |  |
| 32 | n | N/a | yes | stingray | Bonner, J. R., et al. (1983) |
| 26 | m | foot | yes | catfish |  |
| 41 | m | n/a | yes | stingray |  |
| 32 | m | leg | yes | coral | Morris Jr, J. G., et al. (1982) |
| 61 | m | hand | yes | catfish | Clarridge, J. E. and S. Zighelboim-Daum (1985) |
| 38 | m | hand | yes | catfish | Coffey, J. A., et al. (1986) |
| 24 | m | arm + leg | yes | catfish | Hargreaves, J. E. and D. R. Lucey (1990) |
| 10 | m | leg | yes | catfish | Broderick, A., et al. (1985) |
| 23 | m | hand | yes | catfish | Halla, J. T., et al. (1979) |
| 37 | f | foot | yes | stingray | Bendt, R. R. and P. S. Auerbach (1991) |
| 47 | m | foot | yes | stingray | Hønge, B. L., et al. (2018) |
| 31 | m | hand | no | *Plotosus lineatus* | Shepherd, S., et al. (1994) |
| 52 | m | arm | yes | catfish | Huang, G., et al. (2013) |
| 42 | m | hand | no | catfish |  |
| 35 | m | hand | no | catfish | Dorooshi, G. (2012) |
| 76 | m | hand | yes | catfish |  |
| 24 | m | hand | no | catfish | Mann Iii, J. W. and J. R. Werntz (1991) |
| 29 | m | leg | yes | *Pterois volitans* | Moreno, U. L., et al. (2013) |
| 26 | m | leg | yes | *Pimelodus pictus* | Carty, M. J., et al. (2010) |
| 38 | m | hand | yes | catfish |  |
| 59 | f | hand | yes | catfish |  |
| 61 | m | hand | yes | catfish | Baack, B. R., et al. (1991) |
| 62 | m | hand | no | Scorpionfish | Halstead, B. W. (1951) |
| 20 | m | hand | no | *Pterois volitans* | Lucerna, A., et al. (2017) |
| 51 | m | hand | yes | *Netuma thalassinus* | Ortho, M. S. (2019) |
| 31 | m | leg | yes | stingray | Ho, P.-L., et al. (1998) |
| 57 | f | foot | yes | *Synanceia horrida* |  |
| 27 | m | hand | yes | *Synanceia horrida* | Tang, W. M., et al. (2006) |
| 31 | f | foot | yes | stingray | Jarvis, H. C., et al. (2012) |
| 31 | m | hand | no | *Synanceia sp* | Tay, T. K. W., et al. (2016) |
| 50 | m | hand | no | Stonefish | Ling, S. K. K., et al. (2009) |
| 52 | f | leg | no | *Synanceia horrida* | Brenneke, F. and C. Hatz (2006) |
| 51 | m | foot | yes | Stingray |  |
| 67 | m | foot | yes | Stingray | Torrez, P. P. Q., et al. (2015) |
| 37 | m | foot | yes | *Synanceia verrucosa* | Issack, M. I., et al. (2008) |
| various | various | various | yes x8 | Stingray |  |
| various | various | various | no x14 | Stingray | Cevik, J., et al. (2022) |
| 24 | m | hand | no | *Pterois volitans* | Badillo, R. B., et al. (2012) |
| various | various | various | no x 45 | Lionfish |  |
| various | various | various | no x 6 | *Scorpaena guttata* |  |
| various | various | various | yes x 4 | mixed | Kizer, K. W., et al. (1985) |
| 47 | f | foot | no | Scorpionfish | Darlene, F. O. and C. Phee-Kheng (2013) |
| various | various | various | no x 13 | *Pterois volitans* |  |
| various | various | various | yes x 2 | *Pterois volitans* | Haddad, V., et al. (2015) |
| various | various | various | no x 13 | *Scorpaena plumieri* |  |
| various | various | various | no x 8 | *Scorpaena brasiliensis* |  |
| N/a | N/a | N/a | no x 1 | Scorpionfish |  |
| N/a | N/a | N/a | yes x1 | Scorpionfish | Haddad Jr, V., et al. (2003) |
| various | various | various | yes x 8 | Stingray |  |
| various | various | various | no x 111 | Stingray | Clark, R. F., et al. (2007) |
| 49 | m | hand | no | Stingray | Trickett, R., et al. (2009) |
| 12 | m | body | no | Stingray | Caceres, A., et al. (2020) |
| 18 | m | leg | no | Stingray | Negreiros de Holanda, M., et al. (2019) |
| N/a | f | body | no | *Hapalochlaena maculosa* | Edmonds, C. (1969) |
| 4 | m | hand | no | *Hapalochlaena sp* | Cavazzoni, E., et al. (2008) |

**Supplementary table** ***8:*** *Data table of known offender of infection following snake envenoming in the review*

| Bacteria | Animal | Source |
| --- | --- | --- |
| *Klebsiella aerogenes* |  |  |
| *Pseudomonas aeruginosa* | Rattle snake | [Gelman, D., et al. (2022).](https://pubmed.ncbi.nlm.nih.gov/34049730/) |
| *Enterococcus faecalis x10* |  |  |
| *Schewanella putrefaciens x3* |  |  |
| *Serratia marcescens x1* |  |  |
| *Bacteroides fragilis x3* |  |  |
| *Morganella morganii x7* | *Naja atra* | Tsai, Y.-H., et al. (2017). |
| *Pasteurella multocida* | Viper | [Nadiyah, A., et al. (2015).](https://europepmc.org/article/MED/33557450) |
| *Providencia rettgeri* | Snake | Cheong, C. Y., et al. (2010). |
| *Aeromonas hydrophila x3* | Snake | Jorge, M. T., et al. (1998) |
| *Escherichia coli* |  |  |
| *Staphylococcus aureus* | *Crotalus durissus collilineatus* | Nishioka, S. d. A., et al. (2000). |
| *Schewanella putrefaciens x10* | Cobra | Liu, P.-Y., et al. (2012). |
| *Proteus vulgaris* | Cobra | [Saaiman, E. L. and P. J. Buys (2022).](https://pubmed.ncbi.nlm.nih.gov/36472321/) |
| *Pseudomonas aeruginosa* |  |  |
| *Morganella morganii* |  |  |
| *Staphylococcus aureus x2* |  |  |
| *Enterococcus x2* |  |  |
| *Bacteroides fragilis* | Viper | Cheng, C.-L., et al. (2017). |
| *Aeromonas hydrophila* | Viper | Kallel, H., et al. (2018). |
| *Pseudomonas aeruginosa* | Viper | Balaji, G., et al. (2015). |
| *Aeromonas sobria* | Cobra | Le, H. Q., et al. (2021). |
| *Pseudomonas aeruginosa* | Viper | Kaur, H. and G. Mahajan (2018). |
| *Serratia marcescens* | Snake | Chidambaram, M., et al. (2022). |
| *Morganella morganii* |  |  |
| *Enterococcus faecalis* | Cobra | Hearn, P., et al. (2015). |
| *Staphylococcus auerus x 17* |  |  |
| *Enterococcus faecalis x 4* |  |  |
| *Staphylococcus spp x 5* |  |  |
| *Escherichia coli x 8* |  |  |
| *Klebsiella pneumoniae x 4* |  |  |
| *Proteus spp x 3* |  |  |
| *Morganella morganii x 3* |  |  |
| *Pseudomonas aeruginosa x 3* |  |  |
| *Acinetobacter spp x 2* |  |  |
| *Enterobacter spp x 2* | Snake | Garg, A., et al. (2009). |
| *Shewanella algae* |  |  |
| *Morganella morganii* | *Naja atra* | Huang, W.-H., et al. (2021). |
| *Shewanella algae* | *Naja atra* | Liu, P.-Y., et al. (2014). |

**Supplementary table** ***9:*** *Data table of known offender of infection following spider envenoming in the review*

| Infection | Animal | Source |
| --- | --- | --- |
| *Saksenaea vasiformis* (fungus) | *Loxosceles laeta* | [Tormos, E. F., et al. (2019).](file:///C:\Users\leona\Desktop\PhD%20lit%20review\Envenomations\Spiders\1-s2.0-S0363502318303095-main.pdf) |
| *Streptococcus* sp |  |  |
| *Escherichia coli* |  |  |
| *Staphylococcus aureus* |  |  |
| *Proteus* sp. | *Loxosceles* sp. | Majeski, J. (2001). |
| *Apophysomyces elegans* (fungi) | *Loxosceles* sp. | Saravia‐Flores, M., et al. (2010). |

**Supplementary table *10:*** *Data table of known offender of infection following scorpion envenoming in the review*

| Infection | Animal | Source |
| --- | --- | --- |
| *Streptococcus* sp (G group) | Scorpion |  |
| *Streptococcus milleri* | Scorpion | [Wheatley Iii, G. H., et al. (2005).](file:///C:\Users\leona\Desktop\PhD%20lit%20review\Envenomations\Scorps\1-s2.0-S0003497504008100-main.pdf) |
| *Streptococcus bovis* | *Leiurus hebraeus* | Sofer, S., et al. (1996). |
| *Saksanaea vasiformes* (fungus) | Scorpion | Lechevalier, P., et al. (2008). |
| *Mucor* sp | Scorpion | Pourahmad, M., et al. (2013). |

**Supplementary table 11*:*** *Data table of known offender of infection following hymenopteran envenoming in the review*

| Infection | Animal | Source |
| --- | --- | --- |
| *Staphylococcus pyogenes* | bee | Truskinovsky, A. M., et al. (2001). |
| *Streptococcus* sp |  |  |
| *Pseudomonas aeruginosa* |  |  |
| *Candida albicans* (fungus) | Bee | Richardson, D. and J. P. Schmitz (1997). |
| *Pseudomonas aeruginosa* |  |  |
| *Staphylococcus aureus* |  |  |
| *Enterococcus faecilis* |  |  |
| *Xanthomonas maltophila* | Bee | Anderson, C. R., et al. (1995). |
| *Vibrio vulnificus* | Bee | Liang, J.-H., et al. (2021). |
| Type A *Streptococcus* |  |  |
| Type A *Staphylococcus* | bee | Ryssel, H., et al. (2007). |
| *Staphylococcus aureus* | hornet | Maugeri, R., et al. (2017) |
| Parvovirus | yellowjacket | Can, C., et al. (2021). |
| *Bartonella henselae* | ant |  |
| *Arsenophonus nasoniae* | *Apis mellifera* | Liew, K. C., et al. (2022). |

**Supplementary table** ***12:*** *Data table of known offender of infection following centipede envenoming in the review*

| Infection | Animal | Source |
| --- | --- | --- |
| *Staphylococcus aureus* x2 | Centipede | Uzel, A. P., et al. (2009). |
| *Staphylococcus aureus* | *Scolopendra subspinipes* | Puzzo, A., et al. (2020). |
| *Streptococcus* sp (Group A) | *Scolopendra* sp. | Tanaka, Y., et al. (2022). |

**Supplementary table** ***13:*** *Data table of known offender of infection following aquatic animal envenoming in the review*

| Infection | Animal | Source |
| --- | --- | --- |
| *Photobacterium damselae* | Stingray | Barber, G. R. and J. S. Swygert (2000). |
| *Vibrio alginolyticus* | Octopus | Campanelli, A., et al. 2008 |
| *Pseudomonas oryzihabitans* | *Octopus vulgaris* | Aigner, B. A., et al. (2011). |
| *Enterobacter cloacae x2* |  |  |
| *Staphylococcus epidermidis* |  |  |
| *Staphylococcus aureus* |  |  |
| *Morganella morganii* |  |  |
| *Edwardsiella tarda* | Catfish | Murphey, D. K., et al. (1992). |
| *Vibrio vulnificus* | catfish |  |
| *Vibrio parahaemolyticus* | Stingray | Bonner, J. R., et al. (1983). |
| *Photobacterium damselae* |  |  |
| *Staphylococcus aureus* | catfish |  |
| *Photobacterium damselae* | coral |  |
| *Photobacterium damselae* |  |  |
| *Peptostreptococcus sp* | stingray | Morris Jr, J. G., et al. (1982). |
| *Photobacterium damselae* | Catfish | Clarridge, J. E. and S. Zighelboim-Daum (1985). |
| *Photobacterium damselae* | Catfish | Coffey, J. A., et al. (1986). |
| *Edwardsiella tarda* |  |  |
| *Aeromonas hydrophila* |  |  |
| *Citrobacter freundii* |  |  |
| *Fusobacterium mortiferum* | catfish | Hargreaves, J. E. and D. R. Lucey (1990). |
| *Pseudomonas aeruginosa* | catfish | Broderick, A., et al. (1985). |
| *Mycobacterium terrae* | catfish | Halla, J. T., et al. (1979). |
| *Staphylococcus agalactiae* | stingray | Bendt, R. R. and P. S. Auerbach (1991). |
| *Streptococcus mitis* |  |  |
| *Eikenella corrodens* |  |  |
| *Actinomyces odontolyticus* |  |  |
| *Enterococcus faecalis* | stingray | Hønge, B. L., et al. (2018). |
| *Proteus vulgaris* |  |  |
| *Morganella morganii* | Catfish | Huang, G., et al. (2013). |
| *Edwardsiella tarda* |  |  |
| *Escherichia coli* |  |  |
| *Klebsiella pneumoniae* |  |  |
| *Aeromonas hydrophila* |  |  |
| *Vibrio cholerae* | Catfish | Baack, B. R., et al. (1991). |
| *Staphyoloccus aureus x3* |  |  |
| *Vibrio vulnificus* | stingray | Cevik, J., et al. (2022). |
| *Aeromonas sobria* | *Arius thalassinus* | Ortho, M. S. (2019). |
| *Vibrio alginolyticus* | stingray | Ho, P.-L., et al. (1998). |
| *Vibrio vulnificus* | *Synanceja horrida* | Tang, W. M., et al. (2006). |
| *Staphylococcus viridans* | stingray | Jarvis, H. C., et al. (2012). |
| *Vibrio parahaemolyticus* |  |  |
| *Vibrio cholerae* | *Synanceja verrucosa* | Issack, M. I., et al. (2008). |
